# Generalized Jacobi–Weierstrass operators and Jacobi expansions

**DOI:** 10.1186/s13660-018-1747-2

**Published:** 2018-06-28

**Authors:** José A. Adell, Jorge Bustamante, Juan J. Merino, José M. Quesada

**Affiliations:** 1 Departamento de Métodos Estadísticos, Facultad de Ciencias, Universidad de Zaragoza, Zaragoza, Spain; 2 Facultad de Ciencias Físico-Matemáticas, Benemérita Universidad Autónoma de Puebla, Puebla, México; 3 Departamento de Matemáticas, Universidad de Jaén, Jaén, Spain

**Keywords:** 41A35, 47D06, Jacobi–Weierstrass operators, Abel–Cartwright means, Realization of *K*-functionals, Semi-groups of operators, Fractional powers, Nikolskii–Stechkin type inequality

## Abstract

We present a realization for some *K*-functionals associated with Jacobi expansions in terms of generalized Jacobi–Weierstrass operators. Fractional powers of the operators as well as results concerning simultaneous approximation and Nikolskii–Stechkin type inequalities are also considered.

## Introduction

In this note, we work with two fixed real parameters *α* and *β* satisfying $\alpha \geq \beta\geq-1/2$. We use the following notations:

1 $$ \varrho^{ \alpha,\beta}(x) = (1 - x)^{\alpha}(1 + x)^{\beta}, \quad x \in(- 1, 1), $$

and, for $1 \leq p < \infty$,

$$L^{p}_{(\alpha,\beta)} = \biggl\{ f:[-1,1]\to\mathbb{R}: \Vert f \Vert _{p} = \biggl( \int_{-1}^{1}\bigl| f(x)\bigr|^{p} \varrho^{ \alpha,\beta}(x)\,dx \biggr)^{1/p} < \infty \biggr\} . $$

Moreover, for each $n\in\mathbb{N}_{0}$, $\mathbb{P}_{n}$ is the family of all algebraic polynomials of degree not greater than *n*,

2 $$ w^{\alpha,\beta}_{n} = \frac{(2n + \alpha +\beta + 1)\Gamma(n + \alpha+\beta+ 1)\Gamma(n + \alpha + 1)}{\Gamma(n +\beta + 1) \Gamma(n + 1)(\Gamma(\alpha+ 1))^{2}} $$

(Γ stands for the gamma function) and

3 $$ \lambda_{n}=n(n+\alpha+\beta+1). $$

Since *α* and *β* are fixed, we set *X* for one of the spaces $C[-1, 1]$ or $L^{p}_{(\alpha, \beta)}$.

For $n \in\mathbb{N}$, the Jacobi polynomial $R^{(\alpha,\beta)}_{n}$ is the unique polynomial of degree *n* which satisfies

$$R^{(\alpha,\beta)}_{n} (1) = 1 \quad\text{and}\quad \int_{-1}^{1} Q_{n- 1}(x)R^{(\alpha,\beta)}_{n} (x) \varrho^{ \alpha,\beta }(x)\,dx= 0 $$

for all $Q_{n-1} \in\mathbb{P}_{n- 1}$. We also take $R^{(\alpha,\beta)}_{0} (x) = 1$.

For $f \in X $, the Fourier–Jacobi coefficients are defined by

$$\bigl\langle f,R^{(\alpha,\beta)}_{n} \bigr\rangle = \int_{-1}^{1} f(x)R^{(\alpha,\beta)}_{n} (x) \varrho^{ \alpha,\beta}(x)\,dx,\quad n \in\mathbb{N}_{0}, $$

and the associated expansion is

4 $$ f(x)\sim\sum_{n=0}^{\infty}\bigl\langle f,R^{(\alpha,\beta)}_{n} \bigr\rangle w^{(\alpha,\beta)}_{n} R^{(\alpha,\beta)}_{n} (x). $$

It is known that each $f \in L^{1}_{ (\alpha,\beta)}$ is completely determined a.e. by its Fourier–Jacobi coefficients.

### Definition 1.1

For fixed $\gamma> 0$ and $t > 0$, the generalized Jacobi–Weierstrass kernel is defined by

5 $$ W_{t,\gamma} (x) = \sum_{n=0}^{\infty}e^{{ - t\lambda_{n}^{\gamma}}} w^{(\alpha,\beta)}_{n} R^{(\alpha,\beta)}_{n} (x),\quad x \in[- 1, 1]. $$

For $f \in X $, the generalized Jacobi–Weierstrass (or Abel–Cartwright) operator is defined by

6 $$ C_{t,\gamma} (f, x) = \int_{-1}^{1} \tau_{y}(f, x)W_{t,\gamma} (y)\varrho^{ \alpha,\beta}(y)\,dy,\quad x \in[- 1, 1], $$

where $\tau_{y}(f, x)$ is the translation given in Theorem 2.1 below.

Of course the kernel $W_{t, \gamma}$ and the operator $C_{t, \gamma}$ also depend on *α* and *β* but, for simplicity, we omit these indexes. The (classical) Jacobi–Weierstrass operators correspond to $\gamma= 1$.

The generalized Jacobi–Weierstrass operators have been studied in different papers, but only for parameters satisfying $0 < \gamma\leq 1$. This restriction was considered because in such a case the kernels $W_{t, \gamma}$ are positive and the family $\{C_{t, \gamma}\}$ can be considered as formed by positive operators (see [2, 3], [7], pp. 96–97) and/or as a semigroup of contractions (see [2], pp. 49–52, and [18]). For $\gamma > 1$, one cannot expect the positivity of $W_{t, \gamma}$. For instance, it is known that the analogous generalized Weierstrass kernels for trigonometric expansion are not positive when $\gamma > 1$ (see [6], p. 263).

In this paper we will prove that the operators $C_{t, \gamma}$ can be used as a realization of some *K*-functionals which usually appear in some approximation problems related to Jacobi expansions.

For fixed real $\gamma> 0$, let $\Phi^{\gamma}(X )$ denote the family of all $f \in X $ for which there exists $\Psi^{\gamma}(f) \in X $ satisfying

$$\Psi^{\gamma}(f) (x) \sim\sum_{n=0}^{\infty}\lambda_{n}^{\gamma}\bigl\langle f,R^{(\alpha,\beta)}_{n} \bigr\rangle w^{(\alpha,\beta)}_{n} R^{(\alpha,\beta)}_{n} (x). $$

The associated *K*-functional is defined by

7 $$ K_{\gamma}(f, t)=K_{\gamma}(f, t)_{\alpha,\beta} = \inf_{ g \in \Psi^{\gamma}(X )} \bigl\{ \Vert f - g \Vert _{X} + t \bigl\Vert \Psi^{\gamma}(g) \bigr\Vert _{X } \bigr\} $$

for $f \in X $ and $t > 0$. For different realizations of these *K*-functionals, see [8], Theorem 7.1, and [10], Lemma 2.3. We will not use the characterization of these *K*-functionals in terms of moduli of smoothness. We will show that, for any $\gamma > 0$,

$$\sup_{ 0< s\leq t} \bigl\Vert (I - C_{s,\gamma}) (f) \bigr\Vert _{X} \approx K_{\gamma}(f, t). $$

The notation $A(f, t) \approx B(f, t)$ means that there exists a positive constant *C* such that $C^{-1}A(f, t) \leq B(f, t) \leq CA(f, t)$ with *C* independent of *f* and *t*.

Following [19], for $\gamma > 0$, define

8 $$ (I - C_{t, 1})^{\gamma}= \sum _{j=0}^{\infty}(- 1)^{j} \binom{\gamma}{j} C_{jt, 1}, $$

where

$$\binom{\gamma}{0} = 1 \quad\text{and} \quad\binom{\gamma}{j} = \prod _{k=1}^{j} \frac{\gamma- k + 1}{ k} \quad\text{for } j \in \mathbb{N}. $$

For these operators, we will show the relations

$$K_{\gamma}\bigl(f, t^{\gamma}\bigr) \approx\sup _{ 0< s\leq t} \bigl\Vert (I - C_{s,1})^{\gamma}(f) \bigr\Vert _{X } \approx\sup_{ 0< s\leq t^{\gamma}} \bigl\Vert (I - C_{s,\gamma}) (f) \bigr\Vert _{X }. $$

It is known that, if $Q_{n}$ is a trigonometric polynomial of degree not greater than *n* and $r \in\mathbb{N}$, then

$$\bigl\Vert Q^{(r)}_{n} \bigr\Vert _{p} \leq \biggl(\frac{ n}{ 2 \sin(nh)} \biggr)^{r} \bigl\Vert (1 - T_{h})^{r}(Q_{n}) \bigr\Vert _{p},\quad h \in(0, \pi/n), $$

where $\Vert\cdot\Vert_{p}$ denotes the $L^{p}$-norm of 2*π*-periodic functions and $T_{h}$ is the translation operator. That is, $T_{h}Q(x) = Q(x + h)$. These inequalities are due to Nikolskii [11] and Stechkin [13]. For similar inequalities for algebraic polynomials, see [4] and the references given there. Here we will verify an analogous inequality by considering the operators $\Psi^{r}$ and the linear combination of the Jacobi–Weierstrass operators $C_{t,1}$.

In Sect. 2 we collect some definitions and results which will be needed later. The main results are given in Sect. 3, where the result concerning simultaneous approximation is also included. Finally, in Sect. 4 we present a Nikolskii–Stechkin type inequality.

## Auxiliary results

We need a convolution structure due to Askey and Wainger (see [1]).

### Theorem 2.1

*For each* $h \in[-1, 1)$, *there exists a function* $\tau_{h}: X \to X $ *with the following properties*:

(i) *For each* $f \in X $, *one has*
  $$\Vert \tau_{h} f \Vert _{X } \leq \Vert f \Vert _{X}, \qquad \lim_{ h\to1-} \bigl\Vert \tau_{h}(f) - f \bigr\Vert _{X } = 0 $$
  *and*
  $$\bigl\langle \tau_{h}(f),R^{(\alpha,\beta)}_{n} \bigr\rangle = R^{(\alpha,\beta)}_{n}(h) \bigl\langle f,R^{(\alpha,\beta)}_{n} \bigr\rangle ,\quad n \in\mathbb{N}_{0}. $$
(ii) *For* $f\in X$ *and* $g \in L^{1}_{\alpha,\beta}$, *the integral*
  $$(f * g) (x):= \int_{-1}^{1} \tau_{y}(f, x) g(y) \varrho^{ \alpha,\beta}(y)\,dy $$
  *exists a*.*e*. *in* $[-1.1]$,
  $$f * g = g * f,\qquad f * g \in X,\qquad \Vert f * g \Vert _{p}\leq \Vert g \Vert _{1} \Vert f \Vert _{X} $$
  *and*
  9 $$ \bigl\langle f * g,R^{(\alpha,\beta)}_{n} \bigr\rangle = \bigl\langle f,R^{(\alpha,\beta)}_{n} \bigr\rangle \bigl\langle g,R^{(\alpha,\beta)}_{n} \bigr\rangle ,\quad n \in\mathbb{N}_{0}. $$

For $j > \alpha + 1/2$ and $f \in X $, let

$$S^{j}_{m} (f) = \sum_{k=0}^{m} \frac{A^{j}_{ m-k}}{ A^{j}_{m}} \bigl\langle f,R^{\alpha,\beta)}_{k} \bigr\rangle w^{\alpha,\beta}_{k} R^{(\alpha,\beta)}_{k} (x),\quad A^{j}_{m} = \binom{m+j}{m}, $$

be the *m*th Cesàro means of order *j*. It is known that there exists a constant *C* such that

10 $$ \bigl\Vert S^{j}_{m} \bigr\Vert \leq C, $$

and, for each $f \in X $, one has ([2], Corollary 3.3.3, or [7], Theorem A)

11 $$ \lim_{ m\to\infty} \bigl\Vert f - S^{j}_{m} (f) \bigr\Vert _{X } = 0. $$

We need some classical results related to Banach spaces.

### Definition 2.2

Let *Y* be a real Banach space and $B(Y )$ be the Banach algebra of all bounded linear operators $B: Y \to Y$. A uniformly bounded family of operators $\{T(t): t \geq0\}$ in $B(Y )$ is called an equi-bounded semigroup of class $(C_{0})$ if

12 $$ T(s)T(t) = T(s + t)\quad \text{for }s, t \geq 0,\qquad T(0) = I, $$

and $\lim_{ t\to0+} \Vert f - T(t)f\Vert _{Y} = 0$ for each $f \in Y $.

Let *Y*, $B(Y )$ and $\{T(t): t > 0\}$ be an equi-bounded semigroup as in Definition 2.2. Let $D(Q)$ be the family of all $g \in Y$, for which there exists $Q(g) \in Y$ such that

13 $$ Q(g) = \lim_{ t\to0+} \frac{1}{t} \bigl[T(t) - I \bigr]g $$

(the limit is considered in the norm of *Y*). The operator $Q: D(Q) \to Y$ is called the infinitesimal generator of the semigroup $\{T(t): t \geq0\}$. It is known that *Q* is a closed linear operator and $D(Q) $ is dense in *Y*. For properties of semigroups of operators, see [5].

For $r \in\mathbb{N}$, set

$$D \bigl(Q^{r+1} \bigr) = \bigl\{ f \in Y: f \in D \bigl(Q^{r} \bigr) \text{ and } Q^{r}(f) \in D(Q) \bigr\} $$

and, for $f \in D(Q^{r+1})$,

14 $$ Q^{r+1}(f) = Q \bigl(Q^{r}(f) \bigr). $$

A family of operators $S = \{S_{t},: t > 0\}$, $S_{t} \in B(Y )$ for each $t > 0$ is called a (commutative) strong approximation process for *Y* if, for all $f \in Y$ and $s, t > 0$,

$$S_{s} \bigl(S_{t}(f) \bigr) = S_{t} \bigl(S_{s}(f) \bigr),\qquad \bigl\Vert S_{t}(f) \bigr\Vert _{Y} \leq \Lambda \Vert f \Vert _{Y} \quad\text{and} \quad\lim _{ t\to0+} \bigl\Vert f -S_{t}(f) \bigr\Vert _{Y} = 0, $$

where Λ is a constant. In such a case, we set

$$\theta_{S}(f, t) =\sup_{ 0< s\leq t} \bigl\Vert f - S_{s}(f) \bigr\Vert _{Y}. $$

Let $\phi: [0,1)\to \mathbb{R}^{+}$ be a positive increasing function, $\phi(t)\to0$ as $t \to0$, and $Y_{0}$ be a subspace of *Y*. We say that *S* is saturated with order *ϕ* and with trivial subspace $Y_{0}$ if every $f \in Y$ satisfying

$$\lim_{ t\to0+}\frac{\theta_{S} (f, t)}{\phi(t)} = 0 $$

belongs to $Y_{0}$ and there exists $f \in Y\setminus Y_{0}$ satisfying $\theta_{S}(f, t) \leq C(f)\phi(t)$. The following assertion is known (for instance, see [2], Theorem 2.4.2).

### Theorem 2.3

*Assume that* *Y* *is a Banach space*, $D(B)$ *is a dense subspace of* *Y*, *and* $B: D(B) \to Y$ *is a closed linear operator*. *Let* $S=\{ S_{t}: t > 0\}$ *be a strong approximation process in* *Y* *satisfying* $S_{t}(f) \in D(B)$ *for any* $f \in Y$ *and each* $t > 0$. *If there exists a constant* $\gamma_{0}$ *such that*, *for all* $g \in D(B)$,

15 $$ \lim_{ t\to0+}\biggl\Vert \frac{S_{t}(g) - g}{ t^{\gamma_{0}}} - B(g)\biggr\Vert _{Y} = 0, $$

*then the strong approximation process* *S* *is saturated with order* $t^{\gamma_{0}}$ *and the trivial space is the kernel of* *B*.

## The operators $C_{t, \gamma}$ as a semigroup

In fact, it is known that, for $x \in(-1,1)$, $| R^{(\alpha,\beta)}_{n} (x) |< 1$, [14], pp. 163–164, and there exists a constant *C* such that, for each $n\in\mathbb{N}_{0}$,

16 $$ w^{(\alpha,\beta)}_{n} \leq Cn^{2\alpha+1}. $$

These relations can be used to prove that the series in (5) converges absolutely and uniformly in $[-1, 1]$. Thus $W_{t, \gamma} \in L^{1}_{(\alpha,\beta)}$ and, for each $f \in L^{1}_{ (\alpha,\beta)}$, the series $C_{t, \gamma} (f)$ converges absolutely and uniformly in $[-1, 1]$. Moreover,

$$C_{t, \gamma} (f, x) = (W_{t, \gamma}* f) (x)=\sum _{n=0}^{\infty}e^{{ -t\lambda_{n}^{\gamma}}} \bigl\langle f,R_{n}^{(\alpha,\beta)} \bigr\rangle w_{n}^{(\alpha,\beta)} R_{n}^{(\alpha,\beta)}(x). $$

For these assertions, see [2], p. 30.

Our first result seems to be known. For convenience of the reader, we include a proof.

### Theorem 3.1

*For each* $\gamma > 0$, *the family of operators* $\{ C_{t, \gamma}: t > 0\}$ *is an equi*-*bounded semigroup of operators in* *X*.

*Proof.* It follows from Theorem 3.9 of [15] that the family of operators $\{ C_{t, \gamma}: t > 0\}$ is uniformly bounded.

Condition (12) is derived from the properties of the convolution. In fact, it follows from (9) that, for each $f \in X$ and $k \in\mathbb{N}_{0}$,

$$\begin{aligned} \bigl\langle C_{s+t} (f),R^{(\alpha,\beta)}_{k} \bigr\rangle &= e^{{ -(s + t)\lambda _{n}^{\gamma}}} \bigl\langle f,R^{(\alpha,\beta)}_{k} \bigr\rangle = e^{{ -s\lambda_{n}^{\gamma}}} \bigl\langle C_{t, \gamma} (f),R^{(\alpha,\beta)}_{k} \bigr\rangle \\ &= \bigl\langle C_{s,\gamma} \bigl(C_{t, \gamma} (f) \bigr),R^{(\alpha,\beta)}_{k} \bigr\rangle \end{aligned}$$

and this implies $C_{s+t}(f) = (C_{s,\gamma} \circ C_{t, \gamma}) (f)$.

Finally, for each $k \in\mathbb{N}_{0}$,

17 $$ C_{t, \gamma} \bigl(R^{(\alpha,\beta)}_{k} \bigr) (x) = e^{{ -t\lambda_{n}^{\gamma}}} R^{(\alpha,\beta)}_{k} (x). $$

Hence

$$\lim_{t\to0+} \bigl\Vert R^{(\alpha,\beta)}_{k} - C_{t, \gamma} \bigl(R^{(\alpha,\beta)}_{k} \bigr) \bigr\Vert _{X} = 0. $$

Since the operators $C_{t, \gamma}$ are linear and uniformly bounded and the polynomials are dense in *X*, the last equation holds for every $f \in X$.

Taking into account Theorem 3.1, we denote by $A_{\gamma}$ the infinitesimal generator of $C_{t, \gamma}$ and by $D(A_{\gamma})=D(A_{\gamma}(\alpha, \beta))$ the domain of $A_{\gamma}$. In the next result we give a description of the infinitesimal generator.

### Theorem 3.2

*If* $\gamma, t > 0$ *and* $A_{\gamma}: D(A_{\gamma}) \to X$ *is the infinitesimal generator of* $C_{t, \gamma}$, *then*

$$D(A_{\gamma}) = \Psi^{\gamma}(X)\quad \textit{and} \quad{-} A_{\gamma}(f) = \Psi^{\gamma}(f) $$

*for each* $f \in\Psi^{\gamma}(X)$.

*Moreover*, *for each* $r \in\mathbb{N}$ *and* $f \in D(A^{r}_{\gamma})$,

18 $$ D \bigl(A^{r}_{\gamma}\bigr) = \Psi^{r\gamma} (X)\quad \textit{and}\quad (-1)^{r}A^{r}_{\gamma}(f) = \Psi^{r\gamma} (f), $$

*where* $A^{r}_{\gamma}$ *is defined as in* (14).

### Proof

Since $A_{\gamma}$ is the infinitesimal generator of the semi-group (see (13)), $A_{\gamma}: D(A_{\gamma}) \to X$ is a closed operator.

If $f \in D(A_{\gamma})$, then

19 $$ \bigl\langle A_{\gamma}(f),R^{(\alpha,\beta)}_{n} \bigr\rangle = \lim_{ t\to0+}\frac{ 1}{t} \bigl( e^{{ -t\lambda_{n}^{\gamma}}} - 1 \bigr) \bigl\langle f,R^{(\alpha,\beta)}_{n} \bigr\rangle = -\lambda_{n}^{\gamma}\bigl\langle f, R^{(\alpha,\beta)}_{n} \bigr\rangle . $$

Thus $f\in\Psi^{\gamma}(X)$ and

$$\Psi^{\gamma}(f) = -A_{\gamma}(f). $$

In particular, for each polynomial *P*, one has $P \in D(A_{\gamma})$ and $\Psi^{\gamma}(P) = -A_{\gamma}(P)$.

On the other hand, fix an integer $j > \alpha+ 1/2$. For $f \in\Psi ^{\gamma}(X)$, let $S^{j}_{m} (f)$ and $S^{j}_{m}( \Psi^{\gamma}(f))$ be the *m*th Cesàro means of order *j* of *f* and $\Psi^{\gamma}(f)$, respectively. We know that (see (11))

$$S^{j}_{m} (f) \to f, \quad m \to \infty $$

and

$$-A_{\gamma}\bigl(S^{j}_{m} (f) \bigr) = \Psi^{\gamma}\bigl(S^{j}_{m} (f) \bigr) = S^{j}_{m} \bigl(\Psi^{\gamma}(f) \bigr) \to \Psi^{\gamma}(f). $$

Since $-A_{\gamma}$ is a closed operator, $f \in D(A_{\gamma})$ and $-A_{\gamma}(f) = \Psi^{\gamma}(f)$.

Equations (18) can be proved by recurrence. For instance, (19) can be written as

$$\bigl\langle A^{2}_{\gamma}(f),R^{(\alpha,\beta)}_{n} \bigr\rangle = \bigl\langle A_{\gamma}\bigl(A_{\gamma}(f) \bigr),R^{(\alpha,\beta)}_{n} \bigr\rangle =-\lambda_{n}^{\gamma}\bigl\langle A_{\gamma}(f), R^{(\alpha,\beta)}_{n} \bigr\rangle = \lambda_{n}^{2\gamma} \bigl\langle f,R^{(\alpha,\beta)}_{n} \bigr\rangle . $$

 □

### Theorem 3.3

(i) *If for* $\gamma, t > 0$, *and* $f \in X$

$$\theta_{\gamma}(f, t)= \theta_{\gamma}(f, t)_{\alpha,\beta} = \sup _{ 0< s\leq t} \bigl\Vert (I - C_{s,\gamma} ) (f) \bigr\Vert , $$

*and* $K_{\gamma}(f,t)$ *is defined by* (7), *then*

$$\theta_{\gamma}(f, t) \approx K_{\gamma}(f, t). $$

(ii) *The strong approximation process* $\{ C_{t, \gamma}; t > 0\}$ *is saturated with order* *t* *and the trivial class consists of the constant functions*.

### Proof

(i) From Theorem 3.2 we know that $-\Psi^{\gamma}$ is the infinitesimal generator of $\{C_{t, \gamma}\}$ and $D(A_{\gamma}) =\Psi^{\gamma}(X)$. Thus, the result is a simple consequence of [17], Theorem 1.1, or [5], p. 192.

(ii) We will derive the result from Theorem 2.3, with $B =\Psi^{\gamma}$ and $D(B) = D(A_{\gamma})$. We should verify that $C_{t, \gamma} (f) \in D(A_{\gamma})$ for any $f \in X $ and each $t > 0$.

For any $f\in X$, the Fourier–Jacobi coefficients of *f* are bounded by $\Vert f\Vert_{L^{1}_{(\alpha,\beta)}}$. Taking into account (16), for every $x\in[-1,1]$,

$$\begin{aligned} &\Biggl\vert \sum_{n=1}^{\infty}\lambda_{n}^{\gamma}\exp\{ -t\lambda_{n}\} \bigl\langle f,R^{(\alpha,\beta)}_{n} \bigr\rangle w^{(\alpha,\beta)}_{n} R^{(\alpha,\beta)}_{n} (x) \Biggr\vert \\ &\quad\leq \Vert f \Vert _{L^{1}_{(\alpha,\beta)}} \sum_{n=1}^{\infty}\lambda_{n}^{\gamma}\exp \bigl\{ -t\lambda_{n}^{\gamma}\bigr\} w^{(\alpha,\beta)}_{n} \\ &\quad\leq C \Vert f \Vert _{L^{1}_{(\alpha,\beta)}} \sum_{n=1}^{\infty}\lambda_{n}^{\gamma}\exp \bigl\{ -t\lambda_{n}^{\gamma}\bigr\} n^{2\alpha+1} < \infty. \end{aligned}$$

Since the series converges absolutely and uniformly, it defines a function $g_{t}\in X$ satisfying

$$\bigl\langle g_{t},R^{(\alpha,\beta)}_{n} \bigr\rangle = \lambda_{n}^{\gamma}\exp \bigl\{ -t\lambda_{n}^{\gamma}\bigr\} \bigl\langle f,R^{(\alpha,\beta)}_{n} \bigr\rangle = \lambda_{n}^{\gamma}\bigl\langle C_{t,\gamma}(f),R^{(\alpha,\beta)}_{n} \bigr\rangle ,\quad n\in\mathbb{N}. $$

By definition of the operator $\Psi^{\gamma}$, $C_{t, \gamma} (f) \in \Psi^{\gamma}(X )$ (Theorem 3.2) and

$$\Psi^{\gamma}\bigl(C_{t, \gamma} (f) \bigr) = g_{t}. $$

We have proved that $C_{t, \gamma} (X) \in D(A_{\gamma})$.

If $g\in\Psi^{\gamma}(X) =D(A_{\gamma})$, by definition of the infinitesimal generator,

$$\lim_{ t\to0+}\biggl\Vert \frac{C_{t,\gamma}(g) - g}{ t} - A_{\gamma}(g)\biggr\Vert _{Y} = 0. $$

If $f \in\Psi^{\gamma}(X )$ and $A_{\gamma}(f)=-\Psi^{\gamma}(f) = 0$, then $\langle f,R^{(\alpha,\beta)}_{n} \rangle = 0$ for all $n \in \mathbb{N}$. Therefore *f* is a constant.

From part (i), if $g \in\Psi^{\gamma}(X )$, then

$$\theta_{\gamma}(g, t) \leq C K _{\gamma}(g, t) \leq C t \bigl\Vert \Psi^{\gamma}(g) \bigr\Vert _{X }. $$

Hence, the family

$$\bigl\{ f \in X: \exists C(f) \text{ such that } \theta_{\gamma}(f, t) \leq C(f) t \bigr\} $$

contains nonconstant functions.

Now, from Theorem 2.3, we know that the strong approximation process $\{ C_{t, \gamma}: t > 0\}$ is saturated with order *t*. □

### Remark 3.4

Some characterizations of the saturation class of the strong approximation process $\{C_{t, \gamma}: t > 0\}$ can be given as in [2], Theorems 5.1.1 and 7.4.1, where the case $\gamma=1$ was considered. When $\gamma > 0$ is not an integer, fractional derivatives should be considered. This task would lead us far from our main topic.

### Remark 3.5

A relation similar to (i) in Theorem 3.3 is asserted in [16], p. 2885, for the discrete case and Gauss–Weierstrass type means

$$\widetilde{W}_{\Omega(n),\gamma} (f,x) =\sum_{n=0}^{\infty}e^{{ -(\Omega(k)/\Omega(n))^{\gamma}}} \bigl\langle f,R_{n}^{(\alpha,\beta )} \bigr\rangle w^{(\alpha,\beta)}_{n} R^{(\alpha,\beta)}_{n} (x), $$

with Ω varying in a specified class of functions. The proof suggested there is different from the one given here (it does not use the semi-group structure). The main argument in [16] is that some abstract Riesz means are equivalent (as approximation processes) to some Gauss–Weierstrass type means. This kind of equivalence can also be derived by using Corollary 5.4 of [9]. Anyway, the arguments of [16] and the proof given here are related because both use [15], Theorem 3.9, to obtain a uniformly bounded family of multipliers. Apart from this, other topics considered here are not connected with [16].

The arguments used in the proof of Theorem 3.2 can be used to derive similar relations concerning the fractional powers of the Jacobi–Weierstrass operators $\{C_{t, 1}\}$.

Recall that $A_{1}: D(A_{1}) \to X$ is the infinitesimal generator of $\{ C_{t, 1}, t > 0\}$. For $\gamma> 0$, let $D((-A_{1})^{\gamma},X )$ be the family of all $f \in X $, for which there exists an element $(-A_{1})^{\gamma}(f) \in X $ satisfying

20 $$ \lim_{ t\to0+}\biggl\Vert (-A_{1})^{\gamma}(f) -\frac{1}{t^{\gamma}} (I - C_{t, 1})^{\gamma}(f)\biggr\Vert _{X } = 0, $$

where $(I - C_{t, 1})^{\gamma}(f)$ is defined by (8). This induces a map

$$\bigl(- A^{1} \bigr)^{\gamma}: D \bigl( \bigl(-A^{1} \bigr)^{\gamma},X \bigr) \to X $$

which is called the fractional power of order *γ* of $- A_{1}$.

### Proposition 3.6

*If* $\gamma> 0$ *and* $(- A_{1})^{\gamma}$ *is the fractional power of order* *γ* *of* $- A_{1}$, *then*

$$D \bigl((- A_{1})^{\gamma},X \bigr) = \Psi^{\gamma}(X ) $$

*and*, *for each* $f \in\Psi^{\gamma}(X )$,

21 $$ \Psi^{\gamma}(f) = \lim_{ t\to0+} \frac{1}{t^{\gamma}} (I - C_{t, 1})^{\gamma}(f) = \lim _{ t\to0+} \frac{1}{t} \bigl( f-C_{t, \gamma} (f) \bigr). $$

### Proof

If *γ* is a positive integer or $| a |< 1$, the Taylor expansion gives

$$(1 - a)^{\gamma}= \sum_{j=0}^{\infty}(-1)^{j} \binom{\gamma}{j} a^{j}. $$

Notice that

22 $$\begin{aligned} \bigl\langle (I - C_{t, 1})^{\gamma}(f),R^{(\alpha,\beta)}_{n} \bigr\rangle &= \sum_{k=0}^{\infty}(-1)^{k} \binom{\gamma}{k} \bigl\langle C_{kt, 1}(f),R^{(\alpha,\beta)}_{n} \bigr\rangle \\ &= \sum_{k=0}^{\infty}(-1)^{k} \binom{\gamma}{k} \bigl\langle W_{kt},R^{(\alpha,\beta)}_{n} \bigr\rangle \bigl\langle f,R^{(\alpha,\beta )}_{n} \bigr\rangle \\ &= \bigl\langle f,R^{(\alpha,\beta)}_{n} \bigr\rangle \sum _{k=0}^{\infty}(-1)^{k} \binom {\gamma}{k} \exp ( - kt\lambda_{n}) ) \\ &= \bigl\langle f,R^{(\alpha,\beta)}_{n} \bigr\rangle \bigl( 1-\exp(-t\lambda_{n}) \bigr)^{\gamma}. \end{aligned}$$

Therefore, if $f \in D((- A_{1})^{\gamma},X )$, then

$$\bigl\langle (-A_{1})^{\gamma}(f),R^{(\alpha,\beta)}_{n} \bigr\rangle = (\lambda_{n})^{\gamma}\bigl\langle f,R^{(\alpha,\beta)}_{n} \bigr\rangle . $$

Hence $f \in\Psi^{\gamma}(X )$ and $(- A_{1})^{\gamma}(f) =\Psi^{\gamma}(f)$.

It is clear that, for each polynomial *P*, one has $P \in D((- A_{1})^{\gamma},X)$ and

$$(- A_{1})^{\gamma}(P) = \Psi^{\gamma}(P). $$

On the other hand, fix an integer $j > \alpha + 1/2$. For $f \in\Psi ^{\gamma}(X )$, let $S^{j}_{m} (f)$ and $S^{j}_{m} (\Psi^{\gamma}(f))$ be the *m*th Cesàro means of order *j* of *f* and $\Psi^{\gamma}(f)$, respectively. From (11), as in the proof of Theorem 3.2, one has $\lim_{ m\to\infty} \Vert S^{j}_{m}(f) - f\Vert_{X } = 0$ and

$$\begin{aligned} \lim_{ m\to\infty} \bigl\Vert \bigl(- A^{1} \bigr)^{\gamma}\bigl(S^{j}_{m} (f) \bigr) - \Psi^{\gamma}(f) \bigr\Vert _{X }& = \lim_{ m\to\infty} \bigl\Vert \Psi^{\gamma}\bigl(S^{j}_{m} (f) \bigr) - \Psi^{\gamma}(f) \bigr\Vert _{X } \\ &= \lim_{ m\to\infty} \bigl\Vert S^{j}_{m} \bigl( \Psi^{\gamma}(f) \bigr) - \Psi^{\gamma}(f) \bigr\Vert _{X} = 0. \end{aligned}$$

It was proved in [19], Theorem 4, that $D((- A_{1})^{\gamma},X)$ is dense in *X* and $(- A_{1})^{\gamma}$ is a closed operator. Hence $f \in D((- A_{1})^{\gamma},X)$ and $(- A_{1})^{\gamma}(f) = \Psi^{\gamma}(f)$.

The last equality in (21) was proved in Theorem 3.2, because $\Psi^{\gamma}$ is the infinitesimal generator of $\{ C_{t, \gamma}, t > 0\}$. □

### Theorem 3.7

*For fixed* $\gamma > 0$, *one has*

$$K_{\gamma}\bigl(f, t^{\gamma}\bigr) \approx \sup _{ 0< s\leq t} \bigl\Vert (I - C_{s,1})^{\gamma}(f) \bigr\Vert _{X } \approx \theta_{\gamma}\bigl(f, t^{\gamma}\bigr) $$

*for each* $f \in X $ *and* $t > 0$.

### Proof

From Theorems 3.1 and 3.2 we know that the family $\{C_{t, 1}, t \geq0\}$ is a semi-group of operators of class $(C_{0})$ with the infinitesimal generator $A_{1} =-\Psi^{1}$. From Theorem 1.1 of [17], we know that, for all $f \in X $ and $t > 0$,

$$\inf_{ g \in D((- A_{1})^{\gamma},X )} \bigl( \Vert f - g \Vert _{X } + t^{\gamma}\bigl\Vert (- A_{1})^{\gamma}(g) \bigr\Vert _{X } \bigr) \approx\sup_{ 0< s\leq t} \bigl\Vert (I - C_{s,1})^{\gamma}(f) \bigr\Vert _{X }, $$

where $(- A_{1})^{\gamma}$ is given as in (20). But it was verified in Proposition 3.6 that $\Psi^{\gamma}(X ) = D((- A_{1})^{\gamma},X )$ and $(- A_{1})^{\gamma}(g) = \Psi^{\gamma}(g)$ for each $g \in\Psi^{\gamma}(X )$.

The equivalence with $\theta_{\gamma}(f, t^{\gamma})$ follows from Theorem 3.3. □

### Remark 3.8

When *γ* is an integer, Theorem 3.7 is similar to the Main Theorem in [18], p. 390, but the authors assumed that the operators are positive (plus other conditions).

### Remark 3.9

The results of Theorem 3.7 allow us to obtain equivalent relations between fractional powers $(I - C_{s,1})^{\gamma}$ and some Riesz means as in Theorem 5.1 of [9].

Some result concerning simultaneous approximation can be derived from the ones given above.

### Theorem 3.10

*If* $\gamma,\sigma$, *and* *t* *are positive real numbers and* $f \in \Psi^{\sigma}(X )$, *then*

$$\begin{aligned} &C_{t, \gamma} (f), (I - C_{t, 1})^{\gamma}(f) \in \Psi^{\sigma}(X ), \\ &\bigl\Vert \Psi^{\sigma}(f) - \Psi^{\sigma}\bigl(C_{t, \gamma} (f) \bigr) \bigr\Vert _{X } \leq C \theta_{\gamma}\bigl( \Psi^{\sigma}(f), t \bigr) \end{aligned}$$

*and*

$$\bigl\Vert \Psi^{\sigma}\bigl((I - C_{t, 1})^{\gamma}(f) \bigr) \bigr\Vert _{X } \leq C \theta_{\gamma}\bigl( \Psi^{\sigma}(f), t^{\gamma}\bigr), $$

*where the constant* *C* *is independent of* *f* *and* *t*.

### Proof

If $f \in\Psi^{\sigma}(X )$ and $n \in\mathbb{N}_{0}$, from (17) we obtain

$$\begin{aligned} \bigl\langle C_{t, \gamma} \bigl( \Psi^{\sigma}(f) \bigr),R^{(\alpha,\beta)}_{n} \bigr\rangle &= \exp \bigl(- t \lambda_{n}^{\gamma}\bigr) \bigl\langle \Psi^{\sigma}(f),R^{(\alpha,\beta)}_{n} \bigr\rangle \\ &= \lambda_{n}^{\sigma}\exp \bigl(- t\lambda_{n}^{\gamma}\bigr) \bigl\langle f,R^{(\alpha,\beta)}_{n} \bigr\rangle = \lambda_{n}^{\sigma}\bigl\langle C_{t,\gamma}(f),R^{(\alpha,\beta)}_{n} \bigr\rangle \end{aligned}$$

and from (22) one has

$$\begin{aligned} \bigl\langle (I - C_{t, 1})^{\gamma}\bigl( \Psi^{\sigma}(f) \bigr),R^{(\alpha,\beta)}_{n} \bigr\rangle &= \bigl( 1- \exp(- t \lambda_{n}) \bigr)^{\gamma}\bigl\langle \Psi^{\sigma}(f);R^{(\alpha,\beta)}_{n} \bigr\rangle \\ &= \lambda_{n}^{\sigma}\bigl( 1 - \exp(- t\lambda_{n}) \bigr)^{\gamma}\bigl\langle f,R^{(\alpha,\beta)}_{n} \bigr\rangle = \lambda_{n}^{\sigma}\bigl\langle (I - C_{t, 1})^{\gamma}(f),R^{(\alpha,\beta)}_{n} \bigr\rangle . \end{aligned}$$

Therefore $C_{t, \gamma} (f), (I - C_{t, 1})^{\gamma}(f) \in \Psi^{\sigma}(X)$,

$$\Psi^{\sigma}\bigl(C_{t, \gamma} (f) \bigr) = C_{t,\gamma} \bigl( \Psi^{\sigma}(f) \bigr) \quad\text{and} \quad\Psi^{\sigma}\bigl((I - C_{t, 1})^{\gamma}(f) \bigr) = (I - C_{t, 1})^{\gamma}\bigl( \Psi^{\sigma}(f) \bigr). $$

Now, from Theorem 3.3 one has

$$\bigl\Vert \Psi^{\sigma}(f) - \Psi^{\sigma}(C_{t, \gamma}) \bigr\Vert _{X} = \bigl\Vert (I - C_{t, \gamma} ) \bigl( \Psi^{\sigma}(f) \bigr) \bigr\Vert _{X } \leq C \theta_{\gamma}\bigl( \Psi^{\sigma}(f), t \bigr), $$

and using Theorem 3.7 we obtain

$$\bigl\Vert \Psi^{\sigma}\bigl((I - C_{t, 1})^{\gamma}(f) \bigr) \bigr\Vert _{X } = \bigl\Vert (I - C_{t, 1})^{\gamma}\bigl( \Psi^{\sigma}(f) \bigr) \bigr\Vert _{X } \leq C \theta_{\gamma} \bigl( \Psi^{\sigma}(f), t^{\gamma}\bigr). $$

 □

## A Nikolskii–Stechkin type inequality

### Theorem 4.1

*For each* $r \in\mathbb{N}$, *there exists a constant* *C*, *depending upon* *r*, *such that*, *for every* $\lambda\geq1$ *and for each polynomial* $P \in\mathbb{P}_{\xi(\lambda)}$,

$$\bigl\Vert \Psi^{r}(P) \bigr\Vert _{X} \leq C \lambda^{r} \sup_{ 0< h\leq1/\lambda} \bigl\Vert (I - C_{h,1})^{r}(P) \bigr\Vert _{X }, $$

*where*

$$\xi(\lambda) = \max \bigl\{ k \in\mathbb{N}_{0}: k(k + \alpha+\beta + 1) < \lambda \bigr\} . $$

### Proof

In this proof the infinitesimal generator of $\{C_{t, 1}: t > 0\}$ is denoted by *A*.

From the proof of Lemma 1 in [12] we know that, given $r \in\mathbb{N}$, there exists a constant $C_{1} = C(r)$ such that, for each $f \in X $ and $t > 0$, there is $g_{t} \in D(A^{r+1})$ satisfying

23 $$\begin{aligned} & \Vert f - g_{t} \Vert _{X } \leq\sup _{ 0< h\leq t} \bigl\Vert (I - C_{h,1})^{r}f \bigr\Vert _{X }, \end{aligned}$$

24 $$\begin{aligned} & \bigl\Vert A^{r+1}(g_{t}) \bigr\Vert _{X } \leq C_{1} \frac{1}{t^{r+1}} \sup_{0< h\leq t} \bigl\Vert (I - C_{h,1})^{r}f \bigr\Vert _{X } \end{aligned}$$

and

25 $$ \bigl\Vert (- A)^{r}(g_{t}) \bigr\Vert _{X } \leq C_{1} \frac{1}{ t^{r}} \sup_{ 0< h\leq t} \bigl\Vert (I - C_{h,1})^{r}f \bigr\Vert _{X }. $$

As in [9], for $\lambda> 0$ and $f \in X $, consider the best approximation

$$E_{\lambda}(f) = \inf \bigl\{ \Vert f - P \Vert _{X }: P \in \mathbb{P}_{\xi(\lambda)} \bigr\} . $$

It was proved there (Theorem 6.1) that there exists a constant $C_{2} = C(r, \alpha, \beta)$ such that, for $\lambda> 0$ and $f \in X $,

26 $$ E_{\lambda}(f) \leq C_{2} K_{r+1} \bigl(f, \lambda^{- r- 1} \bigr), $$

and (Theorem 3.2) for each $Q \in\mathbb{P}_{\xi(\lambda)}$,

27 $$ \bigl\Vert \Psi^{r}(Q) \bigr\Vert _{ X } \leq C_{2} \lambda^{r} \Vert Q \Vert _{ X }. $$

Now, fix $\lambda > 0$ and $P \in\mathbb{P}_{\xi(\lambda)}$. Let $g_{t} \in D(A^{r+1}) = \Psi^{r+1}(X)$ (see (18)) be given as (23)–(25) with $t = 1/\lambda$ and $f = P$.

For $\varepsilon > 0$ and $k \in\mathbb{N}_{0}$, choose

28 $$ q(g_{t}, k) \in\mathbb{P}_{\xi(2^{k}\lambda)} $$

such that

29 $$ \bigl\Vert g_{t} - q(g_{t}, k)) \bigr\Vert _{X} \leq (1 + \varepsilon)E_{2^{k}\lambda} (g_{t}). $$

From (26), (18), and (24) we know that

$$\begin{aligned} \bigl\Vert g_{t} - q(g_{t}, k))\bigr\Vert _{X } &\leq C_{2}(1 + \varepsilon)K_{r+1} \bigl(g_{t}, \bigl(2^{k}\lambda \bigr)^{- r- 1} \bigr) \\ &\leq\frac{C_{2}(1 + \varepsilon)}{ (2^{k}\lambda)^{r+1}} \bigl\Vert \Psi ^{r+1}(g_{t}) \bigr\Vert _{X } \\ &= \frac{C_{2}(1 + \varepsilon)}{ (2^{k}\lambda)^{r+1}} \bigl\Vert A^{r+1}(g_{t}) \bigr\Vert _{X} \\ &\leq\frac{C_{1}C_{2}(1 + \varepsilon)}{ (2^{k}\lambda)^{r+1}}\frac{1}{t^{r+1}} \sup_{ 0< h\leq t} \bigl\Vert (I - C_{h,1})^{r}P \bigr\Vert _{X } \\ &= \frac{C_{1}C_{2}(1 + \varepsilon)}{(2^{k})^{r+1}}\sup_{ 0< h\leq1/\lambda} \bigl\Vert (I - C_{h,1})^{r}P \bigr\Vert _{X }. \end{aligned}$$

On the other hand, from the identity

$$q(g_{t}, 0) - g_{t} = \sum_{k=0}^{\infty}\bigl(q(g_{t}, k) - q(g_{t}, k + 1) \bigr), $$

(28), (27), (29), and (26), one has

$$\begin{aligned} \bigl\Vert \Psi^{r} \bigl(q(g_{t}, 0) - g_{t} \bigr) \bigr\Vert _{X } &\leq\sum_{k=0}^{\infty}\bigl\Vert \Psi^{r} \bigl(q(g_{t}, k) - q(g_{t}, k +1) \bigr) \bigr\Vert _{X } \\ &\leq C_{2}\sum_{k=0}^{\infty}\bigl(2^{k+1}\lambda \bigr)^{r} \bigl\Vert q(g_{t}, k) - q(g_{t}, k + 1) \bigr\Vert _{X } \\ &\leq C_{2} \sum_{k=0}^{\infty}\bigl(2^{k+1}\lambda \bigr)^{r} \bigl( \bigl\Vert q(g_{t}, k) - g_{t} \bigr\Vert _{X } + \bigl\Vert g_{t} - q(g_{t}, k + 1) \bigr\Vert _{X } \bigr) \\ &\leq2C_{1}C_{2}^{2} (1 + \varepsilon) \sup _{ 0< h\leq1\lambda} \bigl\Vert (I - C_{h,1})^{r}P \bigr\Vert _{X } \sum_{k=0}^{\infty}\bigl(2^{k+1}\lambda \bigr)^{r} \frac{1}{(2^{k})^{r+1}} \\ &= 2^{r+1}C_{1}C_{2}^{2} (1 + \varepsilon) \lambda^{r} \sup_{ 0< h\leq1\lambda} \bigl\Vert (I - C_{h,1})^{r}P \bigr\Vert _{X } \sum _{k=0}^{\infty}\frac{1}{2^{k}} \\ &= C_{3}(1 + \varepsilon)\lambda^{r} \sup _{ 0< h\leq1/\lambda} \bigl\Vert (I - C_{h,1})^{r}P \bigr\Vert _{X }. \end{aligned}$$

We also need the inequality (see (18) and (25))

$$\begin{aligned} \bigl\Vert \Psi^{r}(g_{t}) \bigr\Vert _{X } &= \bigl\Vert A^{r}(g_{t}) \bigr\Vert _{X } \leq C_{1} \frac{1}{ t^{r}}\sup_{ 0< h\leq t} \bigl\Vert (I - C_{h,1})^{r}P \bigr\Vert _{X } \\ &= C_{1}\lambda^{r} \sup_{ 0< h\leq1/\lambda} \bigl\Vert (I - C_{h,1})^{r}P \bigr\Vert _{X }. \end{aligned}$$

From the inequalities given above, for $P \in\mathbb{P}_{\xi(\lambda )}$, we obtain

$$\begin{aligned} \bigl\Vert \Psi^{r}(P) \bigr\Vert _{X } &\leq \bigl\Vert \Psi^{r} \bigl(P - q(g_{t}, 0) \bigr) \bigr\Vert _{X} + \bigl\Vert \Psi^{r} \bigl(q(g_{t}, 0) \bigr) \bigr\Vert _{X } \\ &\leq C_{2}\lambda^{r} \bigl\Vert P - q(g_{t}, 0) \bigr\Vert _{X } + \bigl\Vert \Psi ^{r}(g_{t}) \bigr\Vert _{ X } + \bigl\Vert \Psi^{r} \bigl(g_{t} - q(g_{t}, 0) \bigr) \bigr\Vert _{X } \\ &\leq C_{1}\lambda^{r} ( \Vert P - g_{t} \Vert _{X } + \bigl\Vert g_{t} - q(g_{t}, 0) \bigr\Vert _{X_{\alpha,\beta}} + C_{4}\lambda^{r} \sup _{ 0< h\leq1/\lambda} \bigl\Vert (I - C_{h,1})^{r}P \bigr\Vert _{X } \\ &\leq C_{5} \lambda^{r} \sup_{ 0< h\leq1/\lambda} \bigl\Vert (I - C_{h,1})^{r}P \bigr\Vert _{X }. \end{aligned}$$

 □

### Remark 4.2

The problem of obtaining a Nikolskii–Stechkin inequality for fractional derivatives is open.
